# Thermally tunable Dyakonov surface waves in semiconductor nanowire metamaterials

**DOI:** 10.1038/s41598-023-39676-4

**Published:** 2023-07-31

**Authors:** Mostafa Moradi

**Affiliations:** Interdisciplinary Studies Research Institute, Shahid Beheshti University, Tehran, Iran

**Keywords:** Applied optics, Terahertz optics, Metamaterials, Nanowires, Semiconductors, Surfaces, interfaces and thin films

## Abstract

The development of engineered metamaterials has enabled the fabrication of tunable photonic devices capable of manipulating the characteristics of electromagnetic surface waves. Integration of semiconductors in metamaterials is a proven approach for creating thermally tunable metamaterials through temperature control of the semiconductor carrier density. In this paper, an interface consisting of an isotropic dielectric material as a cover and an indium antimonide (InSb) nanowire metamaterial as a substrate, is theoretically introduced to investigate the propagation conditions of Dyakonov surface waves in terahertz (THz) frequencies. Various temperature-dependent properties of Dyakonov surface waves in such a geometry is studied, including allowed THz regions, angular existence domain, dispersion relation, directionality, localization degree and figure of merit. The proposed configuration due to the presence of significant birefringence in InSb nanowire metamaterial, has potential applications in THz sensing, imaging and spectroscopy.

## Introduction

Metamaterials are artificially engineered media that are designed to exhibit extraordinary properties that are not found in naturally existing materials, such as superlensing^1,2^, invisibility^3^, negative permittivity and permeability^4–6^ , waveguiding^7^ and large birefringence^8,9^. Semiconductors are gaining attention to be used in metamaterials due to their distinguished features such as the tunability of properties^10,11^. Small gaps in these types of materials are sensitive to small variations in temperature that leads to significant changes in their optical properties. Furthermore, the frequency range in a semiconductor metamatreial is mostly affected by plasma frequency of the semiconductor, which depends on the free carrier concentration, that is extremely sensitive to the temperature, in contrast to dielectrics and metals. Some semiconductors such as indium antimonide (InSb) have the proper carrier density for their frequency to be located in the terahertz (THz) region, and can therefore be used in place of a metal in conventional metal-dielectric metamaterials^12^. Moreover, semiconductor films or wires can be easily fabricated by vapor deposition techniques and the carrier density of semiconductors can be controlled by doping, laser light, temperature, and external electric and magnetic fields^13–15^.

Electromagnetic surface waves which propagate along the interface of two optically dissimilar media and decay in the perpendecular direction to the interface, play an important role in various areas of science and technology, such as sensing^16^, integrated optics^17^ and microscopy^18^. The well-known surface plasmon polariton (SPP) is an example of an electromagnetic surface wave that propagates along the interface of two media with permittivities of opposite signs, e.g. the interaface between a metal and a dielectric. Such surface waves suffer propagation loss due to the presence of metallic absorbtion in typical SPP configurations. In 1988, Dyakonov theoretically proposed a distinguished kind of surface wave that exists at the interface of two transparent dielectric materials in which at least one of them is an anisotropic uniaxial material^19^. Since the partnering materials in Dyakonov surface waves (DSWs) are lossless dielectrics, they are considered to be lossless surface waves^20^. In comparison with SPPs, DSWs are weakly localized and extremely directional in such a way that in conventional materials DSWs are only available in a very narrow angular range with respect to the optical axis (OA) of the anisotropic medium^21^. Due to this high directionality, first experimental observation of DSWs was reported in 2009, almost two dacades after its first theoretical prediction^22^. After that, more experimental investigations were carried out on DSWs^20,23,24^. DSWs have been studied theoretically in various materials such as biaxial crystals^25,26^, magnetic materials^27^ and metamaterials^28–31^. The Dyakonov plasmon surface wave (DPSW) is a special case of DSW that exists along the boundary between two media which at least one of them is anisotropic and also one component of the permittivity of either media is negative^32–35^. DPSWs have the combination of properties of both the DSWs and the SPPs, for instance, they have both the strong localization degree of SPPs and high directionality of DSWs.

In this paper, a configuration consisting of a semiconductor nanowire metamaterial (NWMM) as a substrate and an isotropic dielectric material as a cover is proposed and the frequency and temperature domains of NWMM permittivities for the propagation of DSWs and DPSWs are studied. Then, the temperature-dependent properties of DSWs and DPSWs such as angular domains of propagation, dispersion curves and the penetration depths are investigated. Finally, the figure of merit (FOM) of the propagation of the surface waves is studied in order to show that the dielectric losses are negigible.

**Figure 1:**
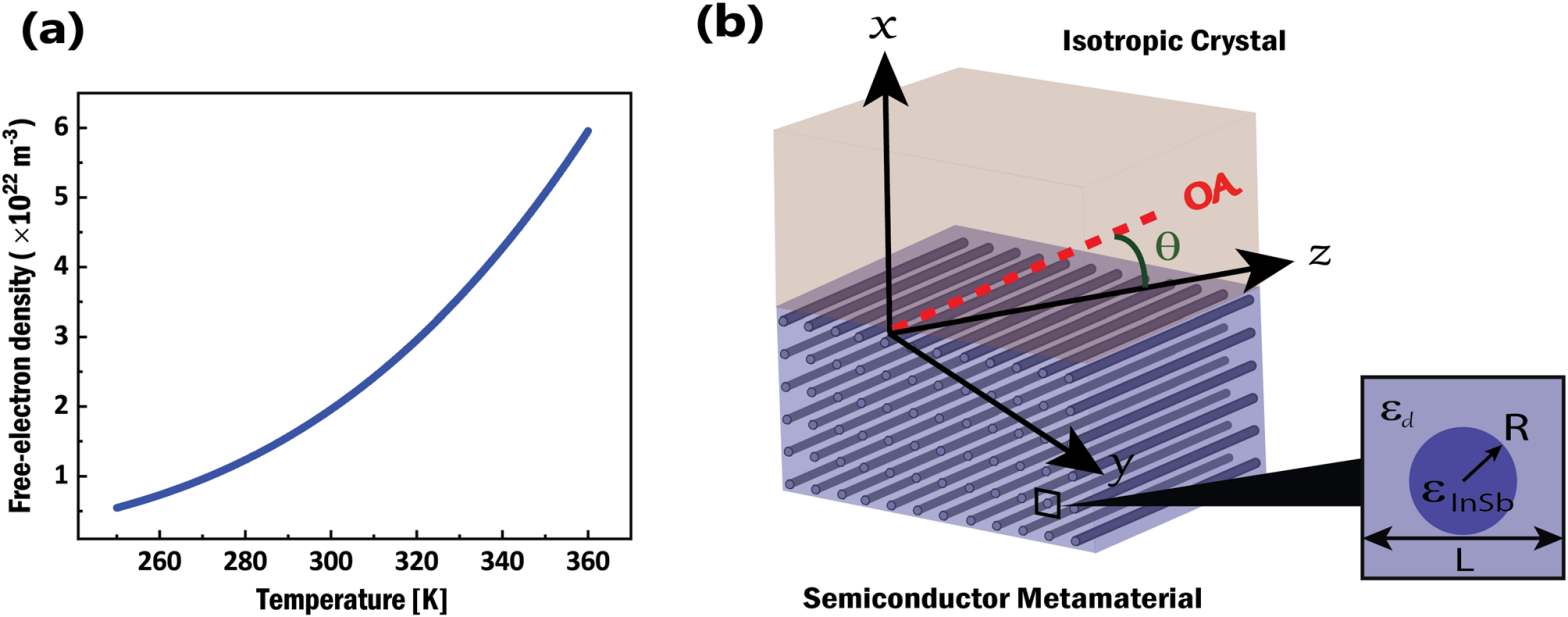
(**a**) The functional relationship between the carrier density of InSb in $m^{-3}$ and temperature in K, (**b**) Schematic of proposed configuration, consisting of an InSb NWMM ($x < 0$) with effective permittivities $\varepsilon _{\parallel }$, $\varepsilon _{\perp }$ and an isotropic dielectric ($x > 0$) with permittivity $\varepsilon _{c}$. Optical axis (OA) of the NWMM lies along the nanowires (y-z plane), and the surface wave propagates along *z* direction forming angle $\theta$ with the OA.

## Configuration and theory

The ability of semiconductors to tune the carrier density and subsequently the permittivity, makes them effective in tunable metamaterials. Among the semiconductors, it is well-known that indium antimonide (InSb) is a great candidate as a thermally tunable material^36–39^. The distinct features of InSb such as the small band gap, low effective mass, high electron mobility and highly temperature-dependent carrier density, make its permittivity easily influenced by a small change in temperature. In the THz frequencies, the complex-valued relative permittivity of InSb is obtained by the Drude model^40^

1 $$\begin{aligned} \varepsilon _{InSb}(\nu )=\varepsilon _{\infty }-\dfrac{\nu _{p}^{2}}{\nu (\nu +i\gamma )} \end{aligned}$$

where $\varepsilon _{\infty }=15.68$ is the high frequency dielectric constant, $\nu$ is the resonant frequency, $\gamma =0.1\pi$ THz is the damping constant and $\nu _{p}=(Ne^{2}/4\pi ^{2}\varepsilon _{0}m^{*})^{1/2}$ is the plasma frequency, where *e* is the electron charge, $m^{*}=0.014m_{e}$ is the effective mass, $m_{e}$ is the electron mass, $\varepsilon _{0}$ is the permittivity of vacuum and *N* is the carrier density in ${\text{m}}^{-3}$ which depends on temperature, *T* (in Kelvin) and in InSb is^41,42^

2 $$\begin{aligned} N=5.76\times 10^{20}T^{3/2}exp(-0.26/2k_{B}T) \end{aligned}$$

where $k_{B}$ is the Boltzmann constant. The variation in *T* causes the change in *N*, which subsequently makes the plasma frequency, $\nu _{p}$ tunable via changing the temperature. The variation of intrinsic carrier denstiy versus temperature is shown in Fig. 1a. When the temperature increases from 250 K to 360 K, the intrinsic carrier density has a significant enhancement from $5\times 10^{21} {\text{m}}^{-3}$ to $6\times 10^{22} {\text{m}}^{-3}$. As it is clear, *N* strongly depends on the temperature, which makes the plasma frequency $\nu _{p}$ tunable by changing the environment temperature. Therefore, in the far-infrared part of the THz region, the permittivity of InSb is very sensitive to the temperature, which makes it a remarkable choice to be used in thermally tunable semiconductor metamaterials.

**Figure 2:**
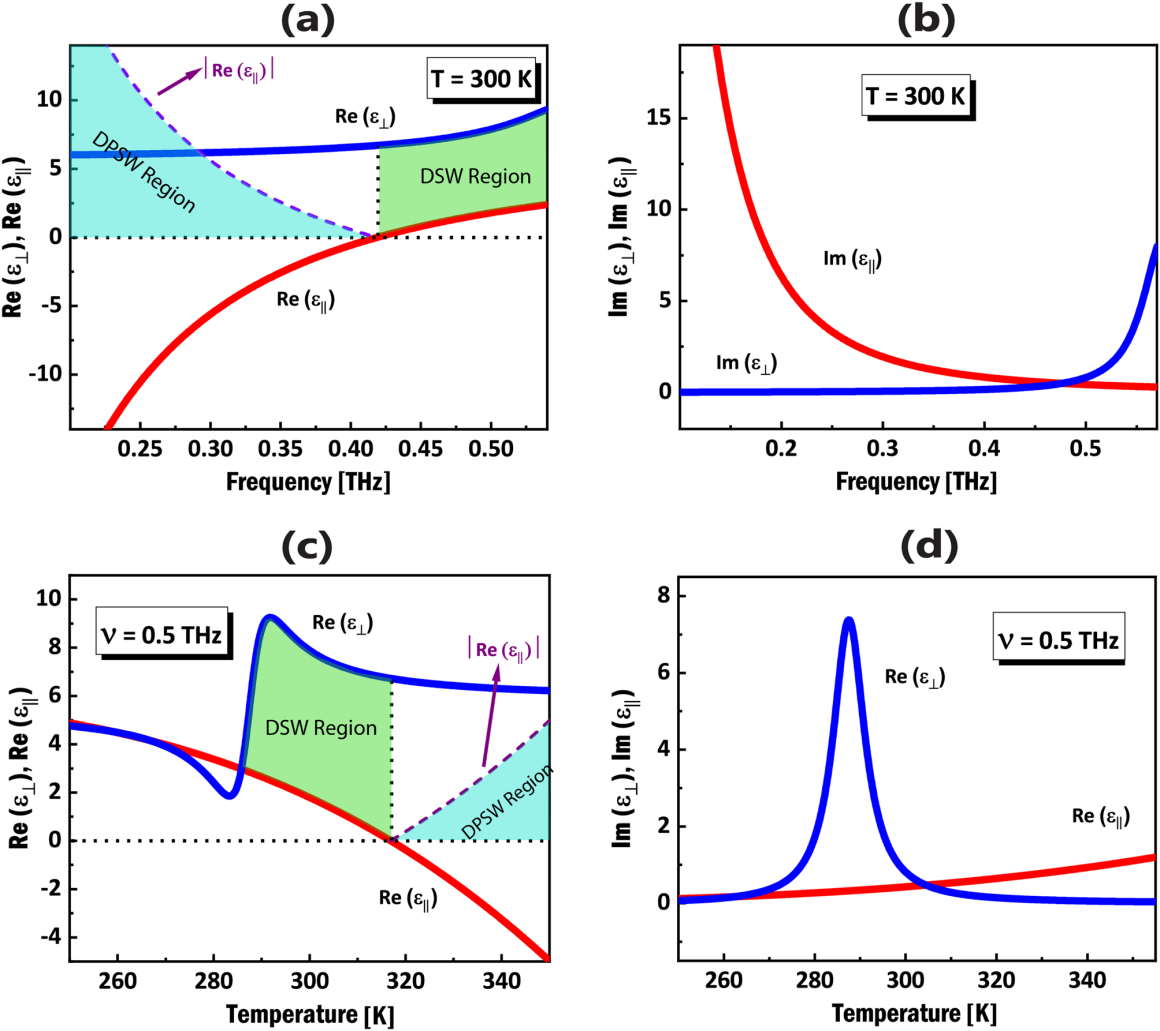
Real and imaginary parts of the effective permittivities $\varepsilon _{\parallel }$ (red line) and $\varepsilon _{\perp }$ (blue line) of the InSb NWMM in (**a**), (**b**) as a function of frequency ($\nu$) at a fixed temperature, $T=300$ K and in (**c**), (**d**) as a function of temperature (*T*) at the fixed THz frequency, $\nu =0.5$ THz. In (**a**), (**c**), the colored regions indicate the necessary condition for the cover permittivity $\varepsilon _{c}$ that supports DSW and DPSW.

Recently nanowire-based composites have attracted significant attention due to their relatively low loss and ease of fabrication^43–47^. Figure 1b illustrates the proposed interface which includes a polycrystalline quartz as an isotropic cover with permittivity $\varepsilon _{c}$ and a semiconductor metamaterial as a substrate. The hyperbolic metamaterial is considered to be nanowires of a semiconductor (InSb) with permittivity $\varepsilon _{InSb}$, embedded into a dielectric host with permittivity $\varepsilon _{d}$. When the nanowire metamaterial (NWMM) parameters, i.e., the nanowire radius (*R*) and the distance between two neighboring nanowires (*L*), are much smaller than the free space wavelength of the incident electromagnetic wave, the system under study can be considered as an effective uniaxial medium with OA parallel to the direction of the nanowires^48^. The permittivity parallel to the nanowires is the ordinary permittivity $\varepsilon _{\parallel }=\varepsilon _{o}$, and the permittivity normal to the nanowires is the extraordinary permittivity $\varepsilon _{\perp }=\varepsilon _{e}$ . On the basis of dynamical Maxwell-Garnett theory, I evaluate the effective permittivities of the NWMM according to^49^

3 $$\begin{aligned} \begin{aligned} \varepsilon _{\parallel }(\nu )&=f\varepsilon _{InSb}(\nu )+\varepsilon _{d}(1-f), \\ \varepsilon _{\perp }(\nu )&=\dfrac{\varepsilon _{d}\varepsilon _{InSb}(\nu )}{\varepsilon _{d}f+(1-f)\varepsilon _{InSb}(\nu )} \end{aligned} \end{aligned}$$

where $\varepsilon _{d}$ is the permittivity of the dielectric host, $\varepsilon _{InSb}$ is the permittivity of the InSb nanowires, and *f* is the semiconductor (InSb) filling ratio, which is defined as

4 $$\begin{aligned} f=\dfrac{\pi R^{2}}{L^{2}} \end{aligned}$$

According to Fig. 1b, *R* and *L* defined in the numerator and denominator in Eq. (4) are nanowire area and unit cell area, respectively. The geometry under investigation consists of a semi-infinite layer of isotropic dielectric cover in the region $x>0$ and a semi-infinite layer of a NWMM in the region $x<0$ (see Fig. 1b). Without loss of generality I consider the case where the OA of the NWMM is in the *zy*-plane.

In the proposed configuration, the permittivity values for vitreous silica as the isotropic cover medium and the $\alpha$-quartz as the dielectric host in InSb NWMM in $\nu =0.5$ THz, are $\varepsilon _{c}=(1.96)^{2}$ and $\varepsilon _{d}=(2.092)^{2}$, respectively^50,51^. Also, the filling ratio of InSb in NWMM is considered to be $f=0.15$. Variation of the real and imaginary parts of the effective permittivities of the InSb NWMM as a function of frequency at temperature $T=300$ K is depicted in Fig. 2a,b. Note that both $\varepsilon _{\parallel }$ and $\varepsilon _{\perp }$, can be complex numbers due to the presence of the dissipation in the Eq. (1). As can be seen, the real part of $\varepsilon _{\perp }$, is always positive in the frequency range 0.2 THz to 0.6 THz, while the real part of $\varepsilon _{\parallel }$, can be either positive or negative, depending on the frequency value. Figure 2c,d illustrate the dependence of NWMM effective permittivities on temperature for a single frequency $\nu =0.5$ THz. The colored area on the right side of Fig. 2a (DSW region in green color) corresponds to the necessary condition for the permittivity of the cover medium in which the pure DSWs exist ($0<\varepsilon _{\parallel }<\varepsilon _{c}<\varepsilon _{\perp }$^19^). In the subsequent calculations for investigating the properties of the DSWs, the frequency $\nu =0.5$ THz is chosen from the frequency interval in the Fig. 2a. The left colored side of the plot (DPSW region in turquoise color) shows the necessary condition for the cover permittivity in which the propagation of the DPSWs are allowed ($0<\varepsilon _{c}<\left| \varepsilon _{\parallel }\right|$^29^). Figure 2a shows that the proposed configuration can support two different types of surface waves in THz frequencies.

The DSWs (and DPSWs) are considered to be hybrid modes which are formed by four evanescent waves with a common wave vector *q*. In the isotropic cover, the field is a superposition of two independent waves of different polarizations (transverse electric, TE and transverse magnetic, TM) with the same wave vector ${{\textbf {q}}}_{{{\textbf {c}}}} = (ik_{c}, ~0, ~q)$ where $k^{2}_{c}=q^{2}-\varepsilon _{c}$. On the other side, in the anisotropic substrate (NWMM), the field is the superposition of the ordinary and extraordinary waves with wave vectors ${{\textbf {q}}}_{{{\textbf {o}}}}=(-ik_{o},~0,~q)$ and ${{\textbf {q}}}_{{{\textbf {e}}}}=(-ik_{e},~0,~q)$ , respectively, where $k_{o}^{2}=q^{2}-\varepsilon _{o}$, $(q^{2}\sin ^{2} \theta -k_{e}^{2})/\varepsilon _{e}+q^{2}\cos ^{2}\theta /\varepsilon _{o}=1$. All of the wave vectors are normalized to $k_{0} = 2\pi \nu /c$ where c is the speed of light in vacuum. By appling the boundary conditions for the tangential components of the electric and magnetic fields at the interface, the dispersion relation of the different kinds of DSWs is obtained (for details see the refs.^34,52^) as follows:

5 $$\begin{aligned} (k_{c}+k_{e})(k_{c}+k_{o})(\varepsilon _{c}k_{o}+\varepsilon _{o}k_{e})= (\varepsilon _{e}-\varepsilon _{c})(\varepsilon _{c}-\varepsilon _{o}) k_{o}. \end{aligned}$$

## Results and discussion

Here I present the results from numerical calculations for the propagation of DSWs and DPSWs in the THz frequencies, at the interface of an isotropic dielectric cover and an InSb NWMM, considering the dispersion relation in Eq. (5). The isotropic cover medium is considered to be vitreous silica with permittivity $\varepsilon _{c}=(1.96)^{2}$ around $\nu =0.5\,\hbox {THz}$^50^. Due to the fact that the effective permittivities of the NWMM strongly depends on the environment temperature (see Fig. 2c,d), I expect the characteristics of DSWs and DPSWs resulting from this NWMM, to be tunable by changing the temperature. The colored areas in Fig. 2a show the THz frequency range for a fixed temperature $T=300$ K in which the propagation of the DSWs and DPSWs are allowed, while the colored areas in Fig. 2c illustrate the temperature range for a fixed frequency $\nu =0.5$ THz.

**Figure 3:**
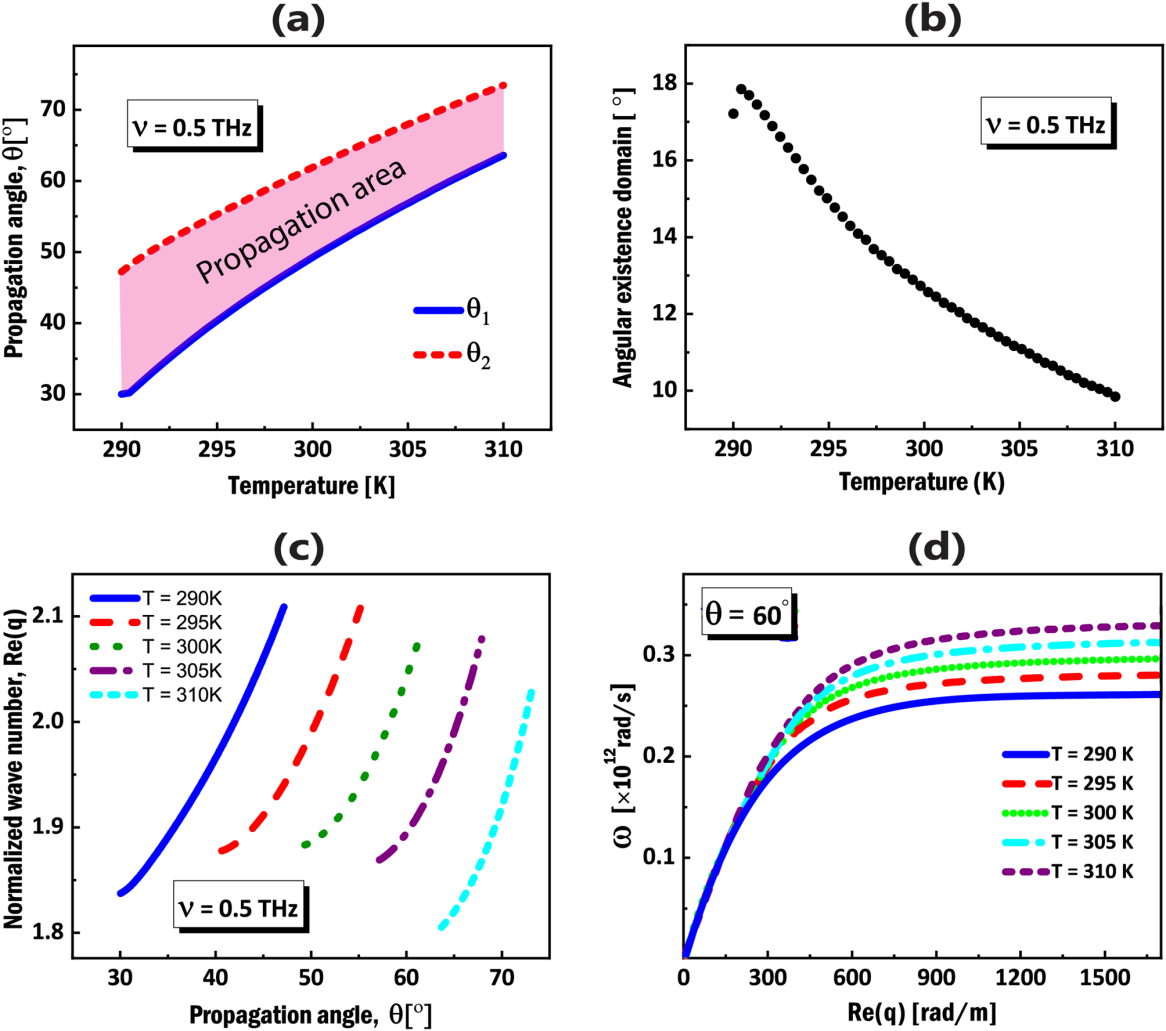
(**a**) Dependence of the propagation angles ($\theta _{1}$, $\theta _{2}$) on the environment temperature at $\nu =0.5$ THz. (**b**) Angular existence domain (AED) of DSWs upon temperature. (**c**) Calculated dispersion relation for DSWs in terms of the propagation angle ($\theta$) for different temperature values. (**d**) Dispersion curves for DPSWs at a fixed propagation angle $\theta =60^{o}$ for different temperature values from $T=290\,\hbox {K}$ to $T=310\,\hbox {K}$.

**Figure 4:**
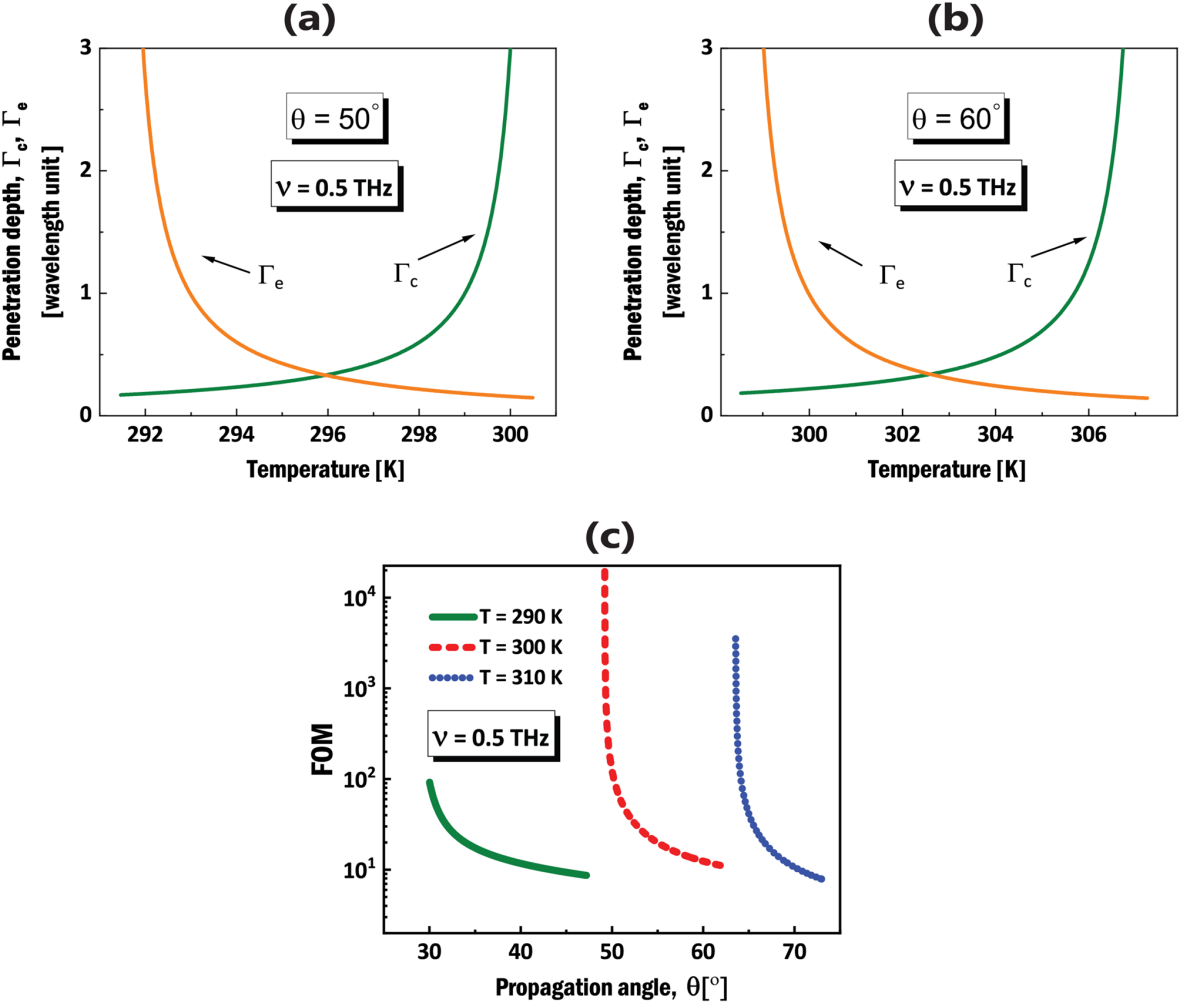
(**a**), (**b**) Penetration depths in the isotropic dielectric cover, $\Gamma _{c}$ (green line) and in the InSb NWMM, $\Gamma _{e}$ (orange line) in terms of the environment temperature for $\theta =50^{o}$ and $\theta =60^{o}$, respectively. (**c**) Figure of merit (FOM) in terms of the propagation angle $\theta$ corresponding to DSWs at the boundary between an isotropic dielectric medium and a lossy InSb NWMM in $\nu =0.5\,\hbox {THz}$.

One of the most challenging obstacles in practical applications of DSWs is the very narrow angular existence domain (AED) of propagation in natural materials^21^. The interval of propagation angle of the DSW with respect to the OA of the NWMM, $\theta _{1}$ and $\theta _{2}$, is derived by, letting $k_{c}=0$ and $k_{e}=0$ in the dispersion relation (Eq. 5), respectively^19^. To study the influence of temperature on the propagation of DSWs, the propagation angles ($\theta _{1}$, $\theta _{2}$) with respect to the OA upon temperature and AED are demonstrated in Figure 3a,b respectively. One can see that in selected temperature interval, the AEDs in the proposed configuration are in the range of 9 to 18 degrees, which are remarkably large, in comparison to that of natural materials^21^. By changing the environment temperature, the AED can be tuned to desired values for practical applications.

Figure 3c demonstrates the dispersion curves of DSWs, considering 5 different temperatures. Using Eqs. (1-4), real parts of the ordinary and extraordinary effective permittivities of the NWMM in $\nu =0.5 \,\hbox {THz}$ are $\varepsilon _{\parallel }=2.66,~ \varepsilon _{\perp }=8.77$, for $T=290 \,\hbox {K}$, $\varepsilon _{\parallel }=2.24, ~\varepsilon _{\perp }=8.77$ for $T=295\, \hbox {K}$, $\varepsilon _{\parallel }=1.80, ~\varepsilon _{\perp }=7.89$ for $T=300\, \hbox {K}$, $\varepsilon _{\parallel }=1.31, ~\varepsilon _{\perp }=7.35$ for $T=305\,\hbox {K}$ and $\varepsilon _{\parallel }=0.79, ~\varepsilon _{\perp }=7.02$ for $T=310\, \hbox {K}$. The dispersion curves are obtained by numerical solving of the wave vector relations for cover and substrate and the dispersion relation (Eq. 5), simultaneously. It is shown in Fig. 3c that in addition to wide angular domains of propagation in all 5 cases, different angular regions, are thermally tunable via changing the temperature values. The plot shows by increasing the temperature, the angular range of DSW propagation shifts to larger values. The angular directionality is one of the distinct features of DSWs that can lead to steering operations of light at the interface^32,53^, and also this feature can be manipulated by changing the temperature, the launching frequency and the cover and the substrate optical parameters.

The DPSW is a special case of DSW that in general, can exist at the interface between two materials which at least one of them is anisotropic and also one component of the permittivity of either media is negative^32–34^. In the present case, the partnering materials have 3 different permittivity values; one for the isotropic cover and two for NWMM, among which the ordinary permittivity of the NWMM is considered to be negative, as was depicted in the colored areas (turquoise color) in Fig. 2a,c. Figure 3d shows the dispersion relation of DPSWs at a fixed propagation angle $\theta =60^{o}$ (with respect to the OA of the NWMM) for different temperatures. The dispersion relation are obtained via implementing the frequency-dependent permittivity of InSb (Eq. (1)) in Eq. (5). It is shown in the plot (Fig. 3d), that DPSWs in different temperatures have different dispersion curves, confirming their tunability upon temperature. Moreover, the specific birefringence of the engineered NWMM for various temperature values, leads to different cut-off frequencies, as is shown in Fig. 3d.

One of the important characteristics of all kinds of surface waves, is the localization degree to the interface. It is well-known that DSWs in natural dielectric materials, are weakly localized to the interface, in comparison to the SPPs that are strongly localized due to the presence of metals. Hence, in Fig. 4a,b I investigate the penetration depths, defined as $\Gamma _{c}=\lambda /2\pi k_{c}$, $\Gamma _{e}=\lambda /2\pi k_{e}$, where $\lambda$, $k_{c}$ and $k_{e}$ are the wavelength and evanescent wave vector components, respectively^28^. The subscripts *c* and *e* represent the isotropic dielectric cover and the InSb NWMM, respectively. Here, I consider two cases of DSWs with different propagation angles with respect to the OA of the NWMM, that share the same THz frequency $\nu =0.5$ THz, for different temperature values. In addition to large confinement factor in both cases, and in cover and substrate, one can see the role of temperature value regarding the magnitude of localization degrees. Figure 4a,b, demonstrate that for both $\theta =50^{o}$ and $\theta =60^{o}$, the penetration depths can be down to half of the $\lambda$ for specific temperature values.

It is worth noting, finally, the permittivity of the $\alpha$-quartz as the dielectric host in InSb NWMM was taken from experimental results in ref.^51^. The same procedure was carried on the vitreous silica as the isotropic cover^50^. Although, for both materials the absorption coefficients are not zero in THz frequencies, I ignored the loss in permittivities for convenience in calculations. According to refs.^54,55^ I consider a figure of merit (FOM), defined by FOM = Re(*q*)/Im(*q*) to determine the influence of absorption losses on the propagation of DSWs. In other word, calculation of the FOM is a method to measure the propagation length of the surface waves at interfaces consisting of lossy materials. In Fig. 4c, one can see that for 3 different temperatures $T=290$ K, $T=300$ K and $T=310$ K in $\nu =0.5$ THz, FOM has extremely high values for specific propagation angles, therefore ignoring the losses in mentioned isotropic materials in my calculations, is totally acceptable.

## Conclusion

A geometry of a semiconductor NWMM as a substrate and an isotropic dielectric cover, supporting temperature-dependent DSWs, is proposed and studied theoretically. Due to the presence of InSb in the NWMM, the conditions for the propagation of DSWs in THz frequencies is shown to be extremely temperature-sensitive. The calculated AED in different temperature values, reaches up to 18 degrees, that is significant compared to that of natural media. In addition, tunability of the angular range of propagation upon temperature in THz frequencies, is demonstrated. According to the dispersion relation (Eq. (5)), another kind of Dyakonov-like surface wave, known as DPSWs is also supported in the proposed structure. It is shown that for DPSWs, different temperature values lead to different dispersion curves with distinct cut-off THz frequencies. It is also pointed out that the localization degree of the DSW strongly depends on the environment temperature. Finally, the FOM of the propagating DSW is calculated to ensure that neglecting losses related to dielectrics in the configuration, is valid. From a practical point of view, despite the slowness of thermal tuning, the result of this work is beneficial for the development of various controlling techniques and real applications of such structures to manipulate the properties of DSWs.

## Data Availability

The datasets used and/or analyzed during the current study are available from the corresponding author on reasonable request.
